# Mechanisms underlying direct actions of hyperlipidemia on myocardium: an updated review

**DOI:** 10.1186/s12944-019-1171-8

**Published:** 2020-02-08

**Authors:** Yu Si Yao, Tu Di Li, Zhi Huan Zeng

**Affiliations:** Department of Cardiovascular Diseases, The First Affiliated Hospital of Guangdong Pharmaceutical University, Guangzhou, Guangdong 510080 People’s Republic of China

**Keywords:** Hyperlipidemia, Cardiac function, Lipid-lowering drug, Heart failure

## Abstract

Hyperlipidemia is a common metabolic disorder and one of risk factors for cardiovascular disease. Clinical studies have shown that hyperlipidemia increases the risk of non-ischemic heart failure, while decreasing serum lipids can reverse heart dysfunction. Apart from indirectly affecting the function of the heart by promoting the development of atherosclerosis, hyperlipidemia also affects the systolic function and cardiac electrophysiological response of the heart directly, which may be related to gradual accumulation of cardiac lipids and consequent systemic oxidative stress, proinflammatory state and mitochondrial dysfunction. However, the mechanism underlying direct effects of hyperlipidemia on the heart are not fully understood. In this review, we provide an updated summary of recent experimental and clinical studies that focus on elucidating the mechanisms of the action of hyperlipidemia on cardiac function, the relationship between heart failure and serum lipids, and protective effects of lipid-lowering drugs on the heart. The exciting progress in this field supports the prospect of guiding early protection of the heart to benefit the patients with chronic hyperlipidemia and familial hyperlipidemia.

## Introduction

Hyperlipidemia indicates abnormally elevated levels of lipids or lipoproteins in the blood due to abnormal fat metabolism or function, and it is caused by dietary disorders, obesity, genetic diseases such as familial hypercholesterolemia (FH) or other diseases such as diabetes [1]. Patients with hyperlipidemia are about twice as likely to develop cardiovascular disease (CVD).

A number of studies have shown that hyperlipidemia, in addition to well-known role in promoting atherosclerosis in the blood vessels, may directly affect the heart, leading to increased ischemia/reperfusion injury and weakened response to cardiac protective interventions such as ischemic preconditioning and post conditioning [2]. In the absence of obvious coronary artery stenosis, long-term hyperlipidemia leads to the accumulation of cardiac lipids and affect cardiac function and electrophysiological activity [3, 4]. Although both the etiology and impact of hyperlipidemia have been widely investigated, its direct effect on the heart and the underlying mechanism are not fully understood.

Therefore, this review aimed to provide an updated summary of recent experimental and clinical studies that focus on elucidating the mechanisms of the action of hyperlipidemia on cardiac function, the relationship between heart failure and serum lipids, and protective effects of lipid-lowering drugs on the heart.

### Mechanisms of the action of lipids on myocardium

A variety of lipids such as triglycerides (TG) and total cholesterol (TC), and high and low density lipoproteins (HDL, LDL) are involved in the regulation of microvascular function. Hypercholesterolemia decreases coronary blood flow reserve and capillary density, induces apoptosis of capillary endothelial cells and ultimately leads to impaired left ventricular (LV) function. It is advocated that hypercholesterolemia may have an impact on the change of membrane lipid bilayer, the regulation of intracellular calcium ions and isoform expression patterns of myosin heavy chain, making the myocardium more sensitive to exogenous damage (such as hemodynamic overload, myocardial ischemia, diabetes) [5]. In particular, HC diet had significant effects on the expression of some crucial proteins in the heart, including Ca^2+^-ATPase (SERCA), ryanodine receptors (RyR) and Na^+^/Ca^2+^ exchangers [6]. Inhibition of SERCA-2 was associated with timely enrichment of TC in cardiac myocardium, and in rabbits fed with HC diet, SERCA-2 mRNA levels decreased within 4 days [7]. Conversely, overexpression of SERCA-2 reduced the mortality of transgenic mice with hemodynamic overload, and maintained cardiac cell function. On the other hand, peroxisome proliferator-activated receptor gamma coactivator 1-alpha (pgc-1) and mitochondrial function recovery are beneficial to cardiac function, while the accumulation of lipids in the myocardium can adversely affect pgc-1 expression and mitochondrial function [8]. Uncoupling protein 2 (UCP2), located in the mitochondrial intima, reduces the synthesis of adenosine triphosphate (ATP) by decoupling the oxidation of the respiratory chain from the phosphorylation of adenosine diphosphate. TC accumulation in heart tissue decreased pgc-1 mRNA levels, and damaged intracellular energy metabolism by aggravating UCP2 expression. Adverse effects on cardiac function were also associated with increased expression of peroxisome proliferator-activated receptor γ (PPARγ) [9]. Overexpression of PPAR in mice could induce dilated cardiomyopathy, due to increased lipid storage and changes in mitochondrial structure [10].

**Fig. 1:**
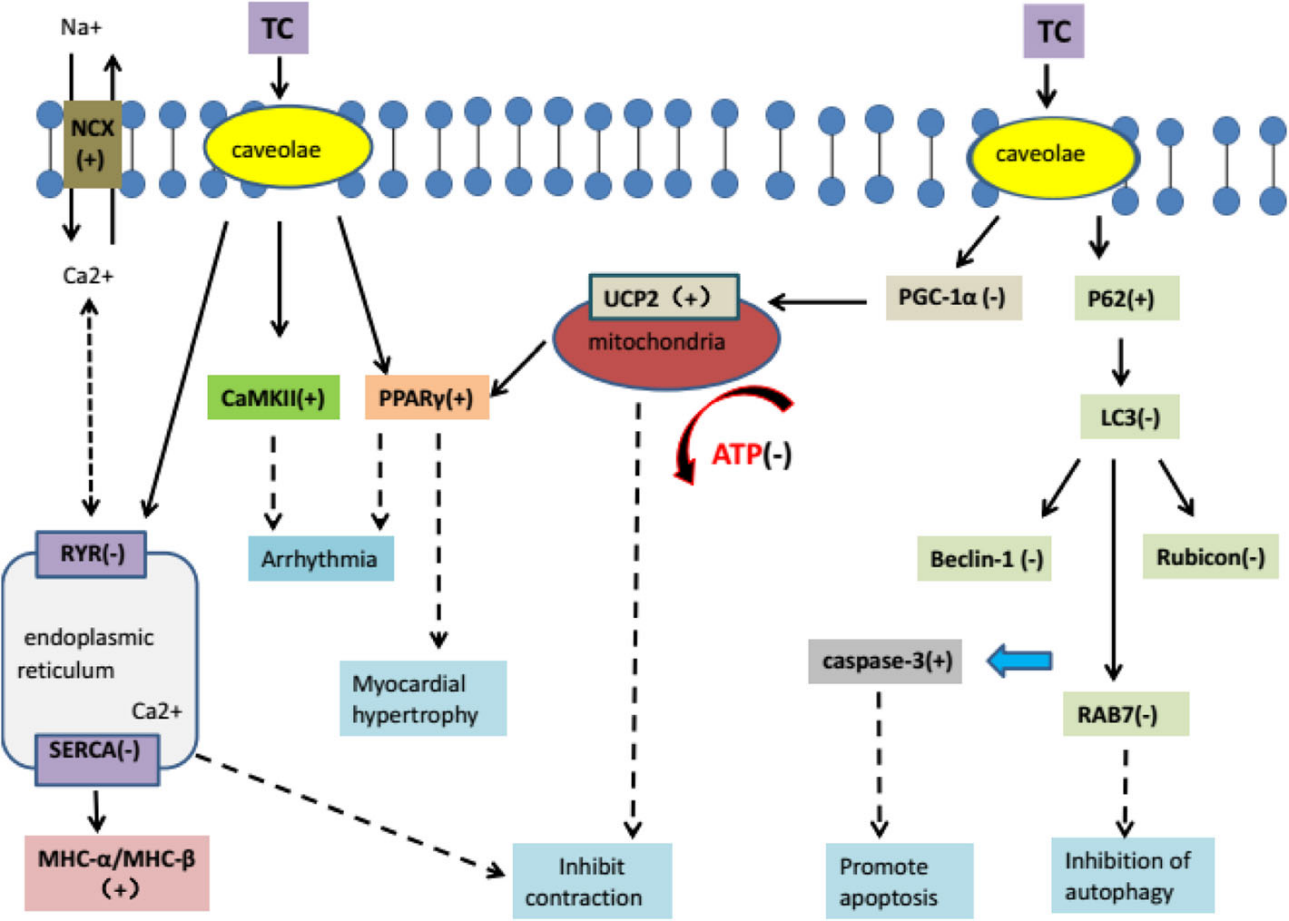
Schematic presentation of the mechanisms of the action of lipids in myocardium. Please see the corresponding text for detailed description

Moreover, hypercholesterolemia may result in myocardial ultrastructure changes through several mechanisms (Fig. 1). First, high-fat and high-cholesterol (HFHC) diet can increase serum TC and free fatty acid (FFAs) levels, leading to systemic oxidative stress and proinflammatory state [11]. Mast cell activation and degranulation promotes inflammation and the release of pro-fibrotic mediators, resulting in tissue fibrosis via transforming growth factor/Wnt/β-catenin pathway [12, 13]. Second, hypercholesterolemia disrupts immune system and induces the production of autoantibodies for G protein coupled receptor, which increase myocardial vulnerability and aggravate heart damage [12]. Third, insufficient autophagy results in apoptosis and cardiac injury [14]. Microtubule-associated protein light chain 3 (LC3) and p62 play an important role in autophagy flux, and hyperlipidemia increased the level of p62 and reduced the expression of LC3 in the heart [15, 16]. Hypercholesterolemia significantly decreased the expression of cardiac autophagy markers but increased the level of cleaved caspase-3, an apoptosis marker in the heart. These results suggest that hypercholesterolemia might inhibit basal cardiac autophagy and promote apoptosis through the mTOR pathway [17].

### Effects of hyperlipidemia on myocardial function

In FH patients, endocardial longitudinal strain (LS), myocardial LS, average LV LS and circumferential strain (CS) decreased significantly, and LDL-c levels were negatively correlated with LS and CS [18]. In addition, heart function was disrupted at an early age in FH patients with high TC levels [19]. Furthermore, LV hypertrophy is more prevalent in diabetic patients with hyperlipidemia than in those suffering from diabetes alone [20].

In animal models of hyperlipidemia, HFHC diet induced cardiac fibrosis and LV diastolic dysfunction in SHRSP5/Dmcr rats [21]. In mice fed with high-fat and high-sugar diet for 8 weeks, LV ejection fraction decreased significantly while isovolumic relaxation time, myocardial performance index and left ventricular end diastolic pressure increased significantly, indicating the damage of both cardiac systolic and diastolic function. Notably, changing to a standard diet partially reversed cardiac contraction and diastolic dysfunction [22]. Moreover, injection of HDL mimetic peptides to hyperlipidemia rabbits for 2 weeks led to significant improvement of LV diastolic function [23].

In conclusion, obesity and hyperlipidemia affect LV structure and function at early stage, and these effects are unrelated to myocardial ischemia and hypoxia caused by coronary heart disease, suggesting that serum lipids affect cardiac function independently of the vascular system, which partly explains high cardiovascular morbidity and mortality resulting from myocardial dysfunction in obesity and hyperlipidemia patients.

### Effects of serum lipids on cardiac electrophysiology

Obesity has been proven as an independent risk factor for arrhythmias in both clinical and experimental studies. Mice with dystrophemia had increased susceptibility to atrioventricular arrhythmia, sympathetic innervation, repolarization dispersion and Ca^2+^ current, along with abnormal expression of gap junction protein and prolonged action potential duration (APD) and QTc interval [24, 25]. In vitro studies showed that isolated adipocytes and free fatty acids directly regulated electrophysiological properties and ionic currents of left atrial and ventricular myocytes, leading to high risk of arrhythmias [26]. Taken together, these in vivo and in vitro data provide strong evidence that obesity or high-fat promotes electrical remodeling and the pathogenesis of arrhythmias. However, little is currently known about the underlying molecular mechanisms.

PPARγ is a crucial transcription factor that regulates lipid metabolism. PPARγ accelerates cellular fat absorption and is upregulated in the heart of patients with metabolic syndrome. Increased lipids and abnormal mitochondrial morphology were observed in the heart of PPARγ transgenic mice [10]. Joseph et al. demonstrated that mitochondrial oxidative stress increased sarcoplasmic reticulum calcium leakage through oxidative RyR2 channel, and ventricular arrhythmia was triggered in mice with lipid overload caused by PPARγ overexpression. In contrast, mitochondria-targeted antioxidants significantly reduced ventricular arrhythmia [27]. Ca^2+^/calmodulin-dependent protein kinase II (CaMK II) not only regulates cardiac electrophysiology and structure, but also plays an important role in various types of arrhythmias [28]. In particular, in mice fed with HF diet, increased expression and activation of CaMKII led to increased sensitivity to arrhythmia-induced electrical remodeling, prolonged action potential duration, downregulated cardiac ion channels including Cav1.2 and Kv4.2/Kv4.3, and decreased conduction velocity (CV). More importantly, all these changes were reversed after treatment with CaMKII inhibitor [29] .

### Relationship between serum lipids and heart failure

FH and high level of non-fasting triglycerides were reported to increase the risk of heart failure [30]. However, low level of serum TC was independently associated with poor prognosis in patients with end-stage heart failure and increased the mortality of ischemic or non-ischemic heart failure [31]. Low HDL and LDL-C levels are closely related to poor prognosis of patients with severe or end-stage heart failure, which is not related to the etiology [32]. In addition, combined reduction of campesterol and lathosterol that indicated intestinal cholesterol absorption and liver synthesis predicted cardiac events, including cardiac-related death, hospitalization for worsening heart failure, and lethal arrhythmia, in patients with mildly symptomatic non-ischemic dilated cardiomyopathy patients [33]. On the other hand, animal study showed that plasma HDL-c and free glycerol levels decreased in lrig3 knockout mice, along with signs of cardiac hypertrophy [34].

**Fig. 2:**
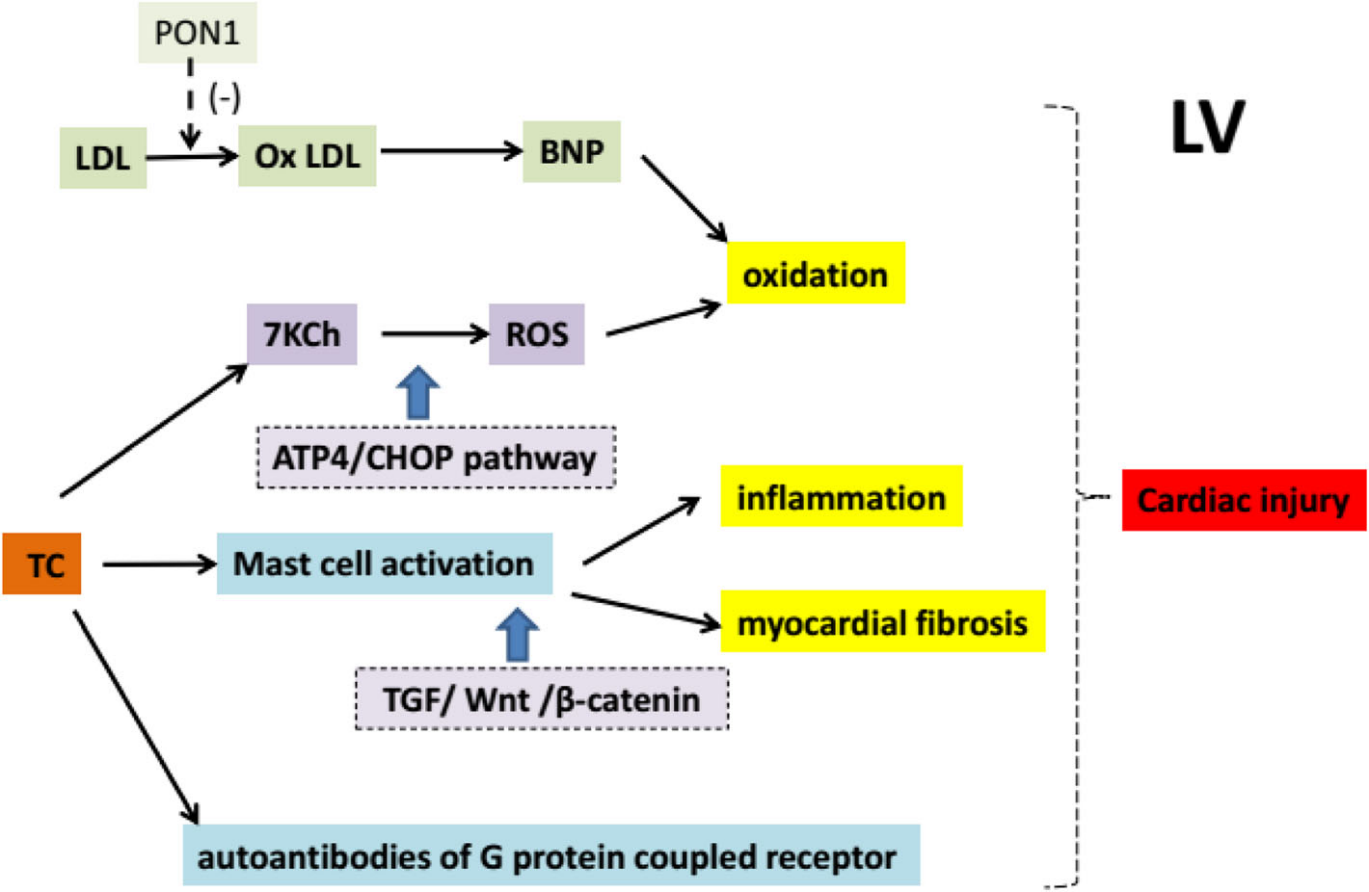
Schematic presentation of several cascades by which serum lipids induce cardiac injury. Please see the corresponding text for detailed description

P-oxyphosphatase 1 (PON1) can inhibit the oxidation of lipids such as LDL [35]. Interestingly, the level of oxidized LDL (Ox LDL) in LV of patients with heart failure was higher and the increase of Ox LDL was correlated with the decrease of ejection fraction (EF) and PON1 activity in LV [36]. Chronic inflammation leads to increased oxidative stress and injury, and is associated with the progression of heart failure [37]. As an oxidative product of TC, 7-ketone cholesterol (7KCh) can induce oxidative stress in cardiomyocytes [38]. A recent study showed that the content of oxidized 7KCh in red blood cells of patients with heart failure was higher than plasma, and the accumulation of 7KCh in cells led to the formation of reactive oxygen species and the death of cardiomyocytes, which may be mediated by ATP4/CHOP pathway [39]. These results indicate that red blood cells may transport 7KCh to heart tissues and cause direct damage to heart cells (Fig. 2). Nevertheless, the causal relationship between increased erythrocyte 7KCh and the development of heart failure remains to be investigated.

### Lowering serum lipids improves heart function

While statins are the most widely used lipid-lowering drugs, the positive effects of statins on the heart go far beyond reducing blood lipids. Furthermore, exploring pharmacological effects of lipid-lowering drugs on the heart may provide important insights into the mechanisms by which hyperlipidemia directly affect the heart. A recent large scale study confirmed that early initiation of lipid-lowering therapy could reduce the incidence of cardiovascular events [40]. In fact, accumulating evidence has suggested that lipid-lowering drugs help improve LV function and inhibit cardiac hypertrophy or remodeling, which is related to the inhibition of vascular inflammation [41].

Atorvastatin improved cardiac function and inhibited LV remodeling in rats with heart failure, via the downregulation of the expression and enzyme activity of matrix metalloproteinase 2 and 9 [42]. In addition, anti-myocardial remodeling effect of statins may benefit from anti-fibrotic mechanism. For example, atorvastatin could attenuate myocardial hypertrophy and remodeling in spontaneously hypertensive rats by inhibiting apoptosis and reversing mitochondrial metabolism via C/EBPβ/PGC-1α/UCP3 signaling pathway [43]. Moreover, pravastatin attenuated cardiac remodeling via inhibiting JNK-dependent pro-apoptotic signaling [44].

Fibrates are fibric acid derivatives that lower blood triglyceride levels and have been used for treating hypertriglyceridemia. Fenofibrate effectively prevented ischemia/reperfusion induced ventricular premature beats, ventricular tachycardia, and ventricular fibrillation in isolated rat hearts [45]. Bezafibrate was reported to reduce myocardial hypertrophy and fibrosis caused by pressure overload via the downregulation of AKT/GSK3β and MAPKs [46]. In addition, gefeizier decreased left ventricular wall thickness via improving cardiac oxidative stress triggered by partial abdominal aortic coarctation [47].

Recently, medicinal plants have attracted more attention in order to screen active compounds with antioxidant and beneficial effects [48, 49]. For example, grape seed procyanidin, an extract with antioxidant, anti-lipid peroxidation and anti-apoptosis properties, could control serum lipid levels close to normal [50]. Hawthorn reduced myocardial fibrosis and heart weight in hyperlipidemic rats by decreasing fasting TG and LDL-C levels [51]. Salvia miltiorrhiza and astragalus miltiorrhiza are common traditional Chinese medicines used to activate blood circulation, and they significantly lower serum lipids and improve cardiac function of patients with coronary heart disease and heart failure [52, 53]. Tongxinluo capsule improved the cardiac function of hyperlipidemic mice by increasing cardiac microvascular density (MVD), and the mechanism of MVD enhancement may be related to the upregulation of vascular endothelial growth factor (VEGF) [54]. These results suggest that anti-VEGF therapy for cancer patients may be detrimental to heart function, and help explain chemotherapy induced hypertension [55]. Most recently, it was shown that supplementation of CoQ10 could reduce myocardial injury by inhibiting p62 and increasing the expression of LC3 in the heart tissue of patients with hyperlipidemia [56].

## Conclusion

Hyperlipidemia is a complex disease that affects heart structure and function even before atherosclerosis occurs. For a long time, the direct effects of serum lipids on cardiac function independent of atherosclerosis are not acknowledged. However, accumulating evidence from recent studies indicates that serum lipids could accumulate in the heart, induce oxidative stress and inflammatory cardiac fibrosis, decrease autophagy and microvascular density, and change the mitochondrial function of cardiomyocytes, making the myocardium vulnerable to damage and leading to cardiac dysfunction and electrophysiological changes. Notably, lowering serum lipid could effectively reverse early ventricular dysfunction and provide heart protection.

However, lipid-lowering drugs have various effects and further studies are needed to focus on the direct effects of these drugs on the myocardium. In addition, the detailed mechanisms and signaling pathways by which lipids induce structural and functional disruption in the myocardium remain to be fully understood. In the clinical, people with hyperlipidemia have no obvious signs or symptoms, but cardiac structure and function may have begun to be damaged. Therefore, powerful diagnosis techniques should be developed to detect these changes at very early stage. Nevertheless, the exciting progress in this field supports the prospect of guiding early protection of the heart to benefit the patients with chronic hyperlipidemia and FH.

## Data Availability

Not applicable.

## Abbreviations

CS: Circumferential strain
CVD: Cardiovascular disease
FFAs: Free fatty acid
FH: Familial hypercholesterolemia
HC: High-cholesterol
HF: High fat
LC3: Light chain 3
LS: Longitudinal strain
LV: Left ventricular
MVD: Microvascular density
pgc-1: Peroxisome proliferator-activated receptor gamma coactivator 1-alpha
PPARγ: Peroxisome proliferator-activated receptor γ
RyR: Ryanodine receptors
SERCA: Ca2 + −ATPase
TC: Total cholesterol
TG: Triglycerides
UCP2: Uncoupling protein 2
